# Fabrication and characterization of 3D-printed PLA-based composite filters for cationic dye removal

**DOI:** 10.1039/d5ra08506c

**Published:** 2025-12-08

**Authors:** Niyazi Erdem Delikanli

**Affiliations:** a Department of Environmental Engineering, Bartın University Bartın 74100 Türkiye edelikanli@bartin.edu.tr

## Abstract

This study presents the fabrication and characterization of 3D-printed biodegradable composite filters for the removal of cationic dyes from aqueous solutions. Inspired by the hierarchical structure of tree leaves, the filters were produced using commercially available polylactic acid (PLA) and a PLA/wood composite filament containing 30% wood dust, *via* fused deposition modeling (FDM). Post-printing modification was performed by treating the filters with aqueous potassium hydroxide (KOH) solutions at varying concentrations (0.1, 0.2, and 0.4 M). The adsorption behavior was assessed using Crystal Violet (CV) as a model cationic dye at concentrations ranging from 10 to 200 mg L^−1^ under gravity driven filtration. KOH treatment significantly enhanced the dye adsorption capacity of PLA/wood composite filters, achieving removal efficiencies up to 97.5%. Surface characterization *via* SEM, FTIR, XRD, and pHpzc analysis confirmed that KOH activation increased surface roughness and introduced functional groups, leading to enhanced electrostatic interaction with cationic species. Moreover, the bioinspired filter geometry with hierarchical porosity contributed to improved mass transfer and dye retention. These findings demonstrate the synergistic effect of material composition, surface modification, and structural design in developing 3D-printed filtration media. The study offers a reproducible and scalable approach for designing polymer-based sorbents for potential applications in water purification.

## Abstract 2

This study presents the fabrication and characterization of 3D-printed biodegradable composite filters for the removal of cationic dyes from aqueous solutions.

## Graphical abstract

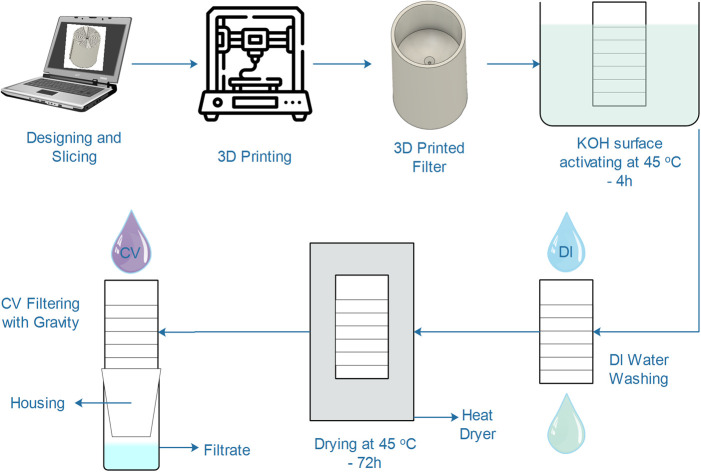

## 1. Introduction

Clean water sources are under increasing global pressure, yet synthetic organic dyes remain difficult to remove with conventional treatment technologies. Owing to their toxic, carcinogenic and highly persistent nature, such dyes pose serious ecological and public-health risks.^1,2^ Crystal Violet (CV)—a widely used cationic dye—is particularly problematic; chronic exposure has been linked to liver and kidney toxicity in animal studies.^3–5^ Conventional removal routes—flocculation, coagulation, electrochemical treatment and advanced oxidation—often suffer from high operating costs and poor efficiency at low dye concentrations.^2,6,7^ By contrast, adsorption onto tailored sorbents offers a simpler and more economical alternative.^8,9^

Recent advances in three-dimensional (3D) printing provide unprecedented freedom to fabricate complex, custom-designed filter geometries from biodegradable feedstocks.^10^ Integrating lignocellulosic wood waste—rich in naturally adsorptive lignin and cellulose—into printed structures can further enhance dye uptake.^11^ Recent reviews (Fahira *et al.*, 2025) confirm the efficacy of wood-based materials for dye removal in aqueous systems, supporting the rationale for incorporating wood-derived fillers into 3D-printed composites.^12,13^ Polylactic acid (PLA), derived from renewable resources such as corn starch, is attractive for this purpose because of its biodegradability and biocompatibility, although its intrinsic brittleness often necessitates composite reinforcement.^14,15^ Wood–PLA composites therefore unite the processability of PLA with the sorptive functionality of lignin and cellulose, while the use of biobased feedstocks aligns with circular-economy manufacturing goals.^16,17^ Moreover, 3D printing enables rapid prototyping and small-scale production, streamlining the optimisation of filter architectures for environmental applications.^18^ Similarly, Delikanlı (2025) demonstrated that natural xylem filters derived from *Pinus pinea* and other woody species could achieve up to 99% turbidity and 95% color removal from river water under gravity driven conditions, confirming the high filtration efficiency of lignocellulosic structures in low-pressure environments.^19^ This natural filtration approach further supports the rationale for incorporating wood-based components into 3D-printed filter matrices to enhance dye removal performance. Recent investigations have also examined the influence of additive-manufacturing parameters on the structural integrity of PLA-based biocomposites. Arslan *et al.* (2025) comprehensively compared injection molding and 3D printing of PLA composites reinforced with basalt fiber and bio-based compatibilizers, demonstrating improved interfacial adhesion and mechanical performance under additive-manufacturing conditions.^20^ These results underline the potential of bio-reinforced PLA systems for functional applications fabricated *via* 3D printing, aligning with the approach adopted in the present work. Kim *et al.* (2020) have already demonstrated the viability of a 3D-printed PLA filter for arsenic removal, underscoring the technology's potential for cost-effective water treatment.^21^

Building on these insights, the present study develops a biodegradable, 3D-printed PLA/wood composite filter for cationic-dye remediation. By optimising material composition and KOH activation, the work aims to deliver an eco-friendly, efficient and reusable filtration platform. Because the filter matrix is PLA-based, its end-of-life fate is also critical. Environmental degradation of PLA proceeds *via* (i) abiotic hydrolysis and (ii) microbial mineralisation. Hydrolysis of ester bonds accelerates sharply above the glass-transition range (∼55–60 °C), fragmenting the polymer into oligomers and lactic acid.^22^ These products are then mineralised by esterase- and lipase-producing microbes to CO_2_ and H_2_O aerobically or to CO_2_, CH_4_ and H_2_O anaerobically. In thermophilic industrial composters (∼58 °C, high moisture, ample O_2_) both steps finish within weeks, and ASTM D6400/EN 13432 tests show ≥60–90% mineralisation in ≤6 months.^23^ Conversely, ambient soil (∼20–30 °C) shows negligible mass or strength loss after 12 months,^24^ while marine samples remain virtually unchanged after 428 days.^25^ Thus, PLA's “biodegradable” label is meaningful only where appropriate composting infrastructure exists; in cool, dry environments the material can persist much like conventional petro-based plastics.

By pairing 3D printing, wood-derived sorbents and controlled KOH activation with an awareness of PLA's realistic degradation pathways, this work seeks to advance a scalable, sustainable strategy for dye-contaminated water treatment. To translate these concepts into a practical design, insights from recent studies on 3D-printed filtration structures were reviewed and adapted to guide the initial configuration of the PLA/wood filter.

In their study on 3D-manufactured filters, Kim *et al.* (2020) stated that differences in channel width would lead to differences in internal surface area and total channel volume.^21^ A smaller, more porous filter with a higher surface area would offer greater filtration capability but less water capacity. Their study demonstrated that a filter with a more comprehensive internal surface area was more effective in adsorptive filtration than one with a smaller surface area. This aligns with the current design, where the spiral shape increases the internal surface area significantly, enhancing the filter's ability to adsorb pollutants effectively. Since the flow rate of water passing through the filter can be controlled by the filter's own architecture, no additional pumping is required. This feature is advantageous for applications in resource-limited areas where additional pumping mechanisms may not be feasible.

Park *et al.* (2023) and Bokseong Kim *et al.* (2023) investigated dye removal using a 3D-printed PLA scaffold filter (grid filter).^26,27^ This filter structure retained contaminants within the liquid *via* an adsorption mechanism rather than acting as a true filter. Lehnen *et al.* (2023) conducted a study on the removal of microorganisms from aqueous media by flow-through using a grid filter.^28^ In their 2021 study, Fijoł *et al.* designed prototypes in the form of 20 × 20 × 20 mm cubes with both gradient and uniform porosity structures, as well as porous disc-shaped models with a diameter of 6 mm, to meet multifunctional requirements and find application in water treatment.^29^ In a subsequent study (Fijoł *et al.*, 2023), four different models were printed and tested for each composite system and reference PLA. These included (i) uniform porosity and (ii) three level gradient porosity cylindrical filters, as well as (iii) uniform porosity and (iv) two level gradient porosity hourglass-shaped filters.^30^ Many of these studies highlight the importance of maximizing surface area for effective contaminant removal. To achieve this, a novel spiral filter design has been developed in this study to maximize contact time and surface area for enhanced dye removal.

Nature offers clear precedents for using spiral geometries to pack a long flow path into a compact space and thereby prolong fluid–surface contact. A well-studied example is the spiral-valve intestine of sharks, skates and rays: a cork-screw mucosal fold winds three to >30 turns inside a short gut segment, markedly slowing digesta transit and multiplying absorptive area; high-resolution CT reconstructions show that this folding functions hydrodynamically as a passive Tesla-type valve, retaining contents far longer than a straight tube of the same length would allow.^31^ A parallel strategy occurs in giant larvaceans (pelagic tunicates), which build gelatinous “houses” whose tightly coiled internal filter tubes force ambient seawater through a helical route that can concentrate pico- and nanoplankton by two to three orders of magnitude before the water exits; *in situ* velocimetry demonstrates that the spiral course, though confined to a house only ∼1 m across, achieves exceptionally long residence times and high filtration efficiencies.^32^ Buoyed by these biological analogues, the 3D-printed spiral filter leverages the same principle—maximising path length and exposure within minimal volume—to enhance water–sorbent interaction and overall purification performance.

## 2. Materials and methods

### 2.1. Filter design & production

**Fig. 1:**
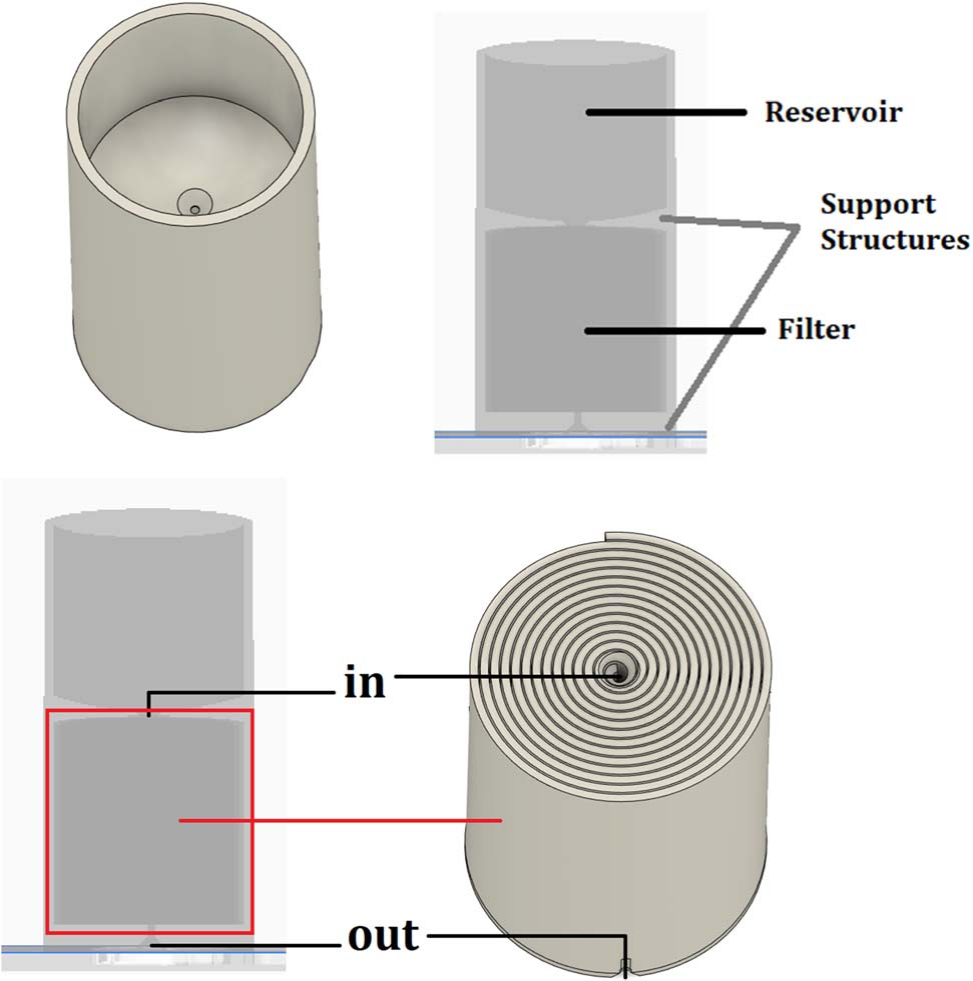
Spiral structure of the 3D-printed filter.

The designed filter is intended to be filled with liquid in the housing section after printing, travel through the spiral structure by gravity, and filter from the other end of the spiral at the bottom. The filter design was made using the Autodesk® Fusion 360 program.^33^ The designed filter is shown in Fig. 1. The total surface area of the water that comes into contact with the designed spiral filter in the design program was calculated as 24 759.27 mm^2^ ≈ 250 cm^2^. If the water had contacted the inner surface of a straight cylinder pipe of the same size, the surface area would have been 114.29 mm^2^ according to the formula π × *D* × *H*. As the distance covered by the water in the filter increases, it is aimed to filter the pollutants in the water by contacting the filter material as much as possible. The total length of the designed part is 69.4 mm, the diameter is 36.25 mm, the effective filter length is 36.4 mm, and the diameter is 32.88 mm. The gap between the housing and the spiral is 1.3 mm, the gap in which the water in the spiral can move is 0.2 mm, and the diameter of the outlet gap from the filter is again 1.3 mm. The housing can hold 23 mL of water. During the design process, various spiral widths, gaps, and reservoir sizes were tested to ensure proper sealing and optimize water circulation within the filter. The first criterion in the design is the space where the water in the spiral will move. The optimum and minimum width produced with a 3D printer is provided as 0.2 mm, and other parameters are calculated according to this parameter. As illustrated in Fig. 1, the 3D-printed filter features a spiral channel to maximize the contact area between the water and the filter material.

**Fig. 2:**
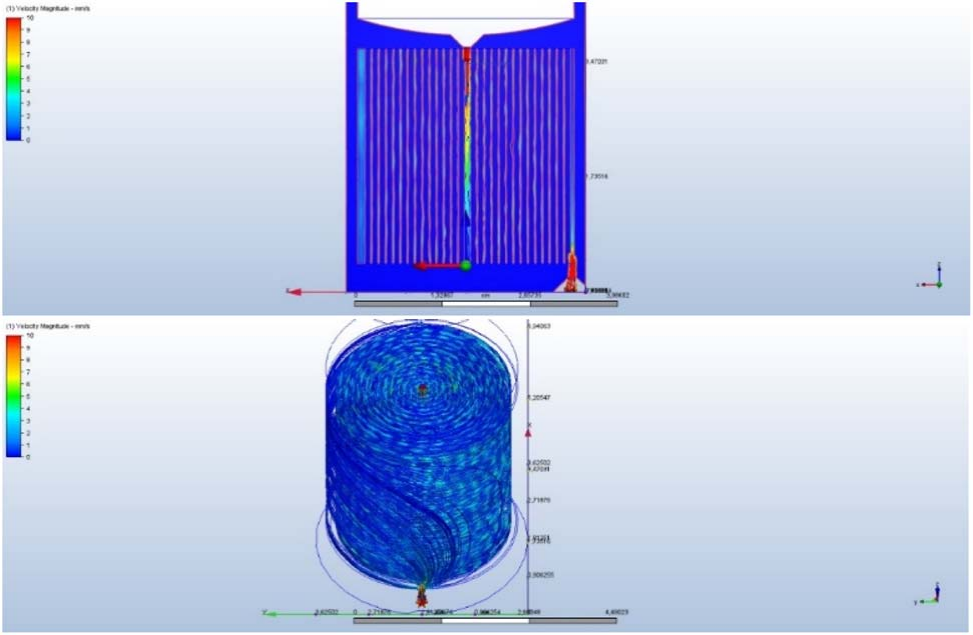
Simulation of water behavior in the filter with CFD analysis.

**Fig. 3:**
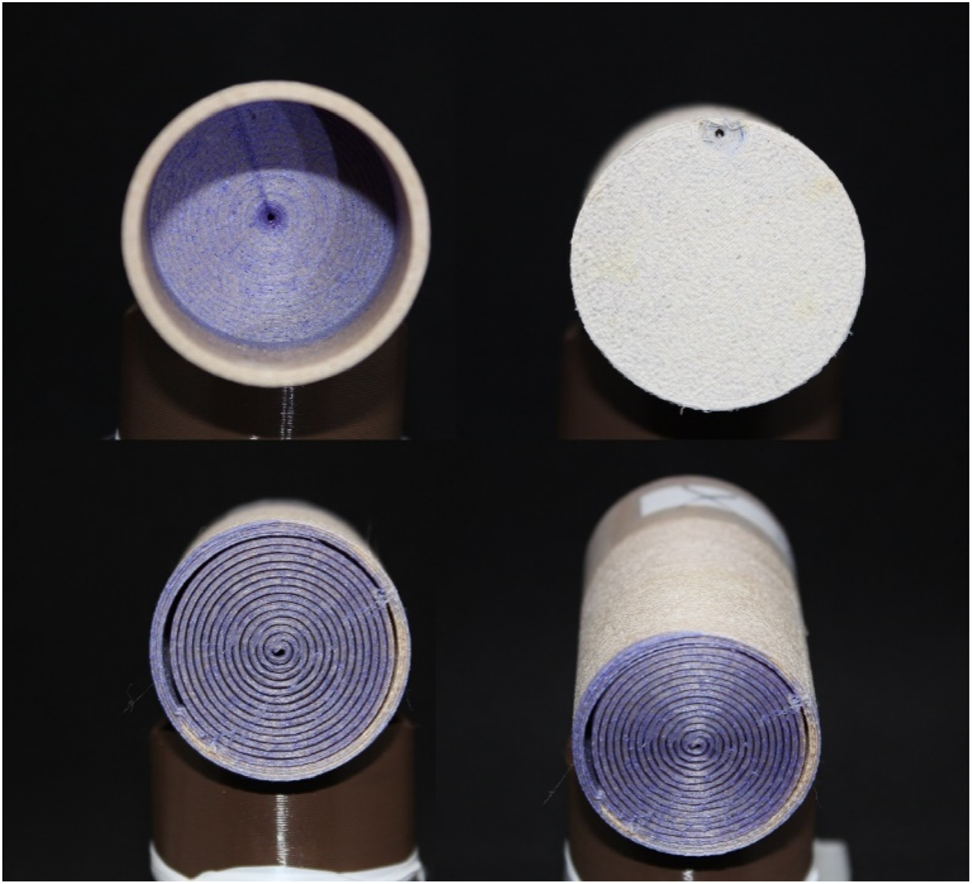
Internal view of the effective area of the designed filter.

Computational fluid dynamics (CFD) simulations were performed using Autodesk® Fusion 360 CFD module (Autodesk Inc., USA). The steady-state, laminar flow model was employed with water as the working fluid (*ρ* = 998 kg m^−3^, *µ* = 1.003 × 10^−3^ Pa s). The inlet velocity was set to 0.05 m s^−1^ under gravity driven conditions, and the convergence criterion was defined as 1 × 10^−4^ for all residuals. The CFD analysis in Fig. 2 confirms that the spiral design of the filter promotes efficient water circulation and maximizes contact with the filter material. To better understand the filter's internal structure, Fig. 3 offers a detailed view of the effective area where the dye removal process occurs.

The housing designed to facilitate the collection of the filtered liquid was produced by 3D printing with Porima Recycle® PLA filament, which enables the reuse of PLA waste materials by converting them into filament. The designed filter comprises a reservoir at the top, a support structure in the middle, a filter at the bottom, and another support structure at the very bottom. The upper part is the reservoir used for holding the dye solution during testing, while the lower part contains the spiral filter and support structures.

This filter housing design is similar to other 3D-printed filtration systems, which often include multiple layers and support structures to enhance stability and efficiency.^34^ Use of recycled PLA filament aligns with recent trends in sustainable manufacturing practices, ensuring that the filter design not only performs effectively but also adheres to eco-friendly principles but also adheres to eco-friendly principles.^35^

The designed filter was prepared for 3D printing by slicing it with Ultimaker® Cura slicing software.^34^ Each filter was designed with a 0.4 mm diameter nozzle and 200–230 °C extruder and printed with a Creality® Ender 3 S-1 3D FDM printer at a bed temperature of 65–70 °C. Printing parameters were set as follows: extrusion temperature 220 °C, bed temperature 65 °C, print speed 50 mm s^−1^, layer height 0.2 mm, and 100% infill density. The print of each filter was completed in approximately 7 hours, and 39 filters were produced for testing. Standard PLA, a biodegradable material, and composite PLA/wood composite (Porima PLA®, Porima PLA Wood®) containing natural wood by weight at a ratio of 30% were used in the study. The technical and mechanical properties of the filaments used are given in Table 1.

**Table 1:** Technical specification table of commercial filaments used (Porima®)

	PLA	PLA/wood	Unit	Test method
**Physical property**				
Density	1.23	n/a^a^	g cm^−3^	ISO 1183
Melt flow index	17.3	12.2	g/10 min	ISO 1183

**Mechanical property**				
Tensile strength	56	45	MPa	ISO 527
Elastic module	2850	3000	MPa	ISO 527
Elongation at break	7	4	%	ISO 527
Notched impact test	14.2	4.2	kJ m^−2^	ISO 179

**Thermal property**				
Heat bending temperature	55	55	°C	ASTM D648
Glass transition temperature	55–60	60–65	°C	ASTM D3418

**Electrical property**				
Surface resistance	>10^12^	>10^12^	Ohm per sq	ASTM D257

a The company (Porima®) does not provide technical information. The density of PLA/wood material is between 0.7 g cm^−3^ and 1.13 g cm^−3^.

The production parameters used in this study are consistent with those reported in the literature for similar applications.^21,34^ The specific printing conditions and parameters, such as nozzle diameter, extruder temperature, and bed temperature, are within the optimal ranges for achieving high-quality prints and ensuring the mechanical integrity of the filters. The choice of PLA and PLA/wood composite materials aligns with current trends in utilizing biodegradable and composite materials for environmental applications.

### 2.2. Surface modification and characterization of 3D-printed filters

In the absence of elevated temperature, KOH acts primarily as a chemical activating agent. It hydrolyses the ester linkages of PLA, yielding carboxylate-terminated oligomers, while simultaneously cleaving covalent bonds within the lignocellulosic matrix (cellulose, hemicellulose and lignin). This treatment increases the surface density of –OH and –COO^−^ groups and partially solubilises wood constituents, thereby promoting the development of macro- and mesoporosity. The concomitant strengthening of aromatic C=C bands in the FTIR spectrum and the overall darkening of the material reflect local aromatisation during this degradation–rearrangement process; yet, in the absence of thermal energy a fully graphitic framework does not form, so the observed changes are better described as KOH-driven surface functionalisation with limited aromatisation rather than classical carbonisation.

Accordingly, KOH-activated wood-filled PLA becomes a hydrophilic, basic and highly porous composite. Partial saponification of the PLA backbone and near-complete dissolution of hemicellulose, together with lignin depolymerisation and rearrangement, generate surfaces rich in –COO^−^/–OH sites, while residual potassium gives rise to K_2_CO_3_/K_2_O templates that further expand micro- and mesopores. The resulting negatively charged surface forms strong electrostatic complexes with cationic dyes such as crystal violet; extended π-domains allow additional π–π stacking, and the overall increase in hydrophilicity reduces diffusion barriers in aqueous media. These combined features furnish the KOH-activated PLA/wood composite with multiple adsorption mechanisms, high specific surface area and abundant basic sites, making it a promising sorbent not only for dye removal but also for heavy-metal sequestration, catalyst support and energy-storage electrodes.

Potassium hydroxide (KOH) is an alkaline base commonly used for surface activation due to its cost-effectiveness and high abrasion rate.^36^ The electrostatic structure of the filter material's surface was modified using KOH to enhance its filtration properties while preserving the mechanical properties of the PLA material. The filters were soaked in different KOH concentrations (0.1 M, 0.2 M, and 0.4 M) at 45 °C for 4 hours to ensure complete surface activation. This temperature was selected based on preliminary tests indicating optimal activation without compromising the filter's structural integrity. In order to remove all remaining KOH, the filters are subsequently washed repeatedly with pure water and then dried at 45 °C for 72 h to achieve complete removal of water along with any other chemicals left on the samples. Potassium hydroxide (KOH) (Merck®), synthetic dye crystal violet (Merck®), Congo red (Merck®), and purified water were used in the experiments. All chemicals were of analytical grade and used as received. To ensure that any residual alkali did not interfere with dye chemistry, all samples were repeatedly rinsed until the filtrate reached neutral pH. Subsequent UV-vis spectra of the filtrates showed no sign of dye bleaching, confirming that KOH acted solely as a surface activator rather than a reactive species toward CV molecules.

Dye removal experiments were carried out at different crystal violet and Congo red concentrations (10, 25, 100, and 200 mg L^−1^) to evaluate the separation capacity of the activated filters. Each experiment involved passing 20 mL of dye solution through the filter at 25 °C. This temperature was chosen to replicate common environmental conditions and ensure consistent results across different experimental setups. The color concentration of crystal violet in the effluent was measured at 586 nm using a UV-vis spectrophotometer (Hach Lange® DR6000).^36,37^ For Congo red, the color concentration was measured at 497 nm using the same spectrophotometer.

Control experiments were conducted using raw filters washed with pure water without KOH treatment. These control filters were tested under the same conditions to provide a baseline for comparison. The results indicated significantly lower dye removal efficiency in the control filters, underscoring the effectiveness of KOH surface activation in enhancing cationic dye separation. The surface chemistry and point of zero charge (pHpzc) of the filter material were characterized using the pH equilibrium method.^38,39^ After 3D printing, the filters were crushed and sieved to <1 mm for pHpzc testing. The pHpzc analysis revealed that the KOH treatment effectively increased the surface negative charge, which facilitated the separation of cationic dyes like crystal violet.

**Fig. 4:**
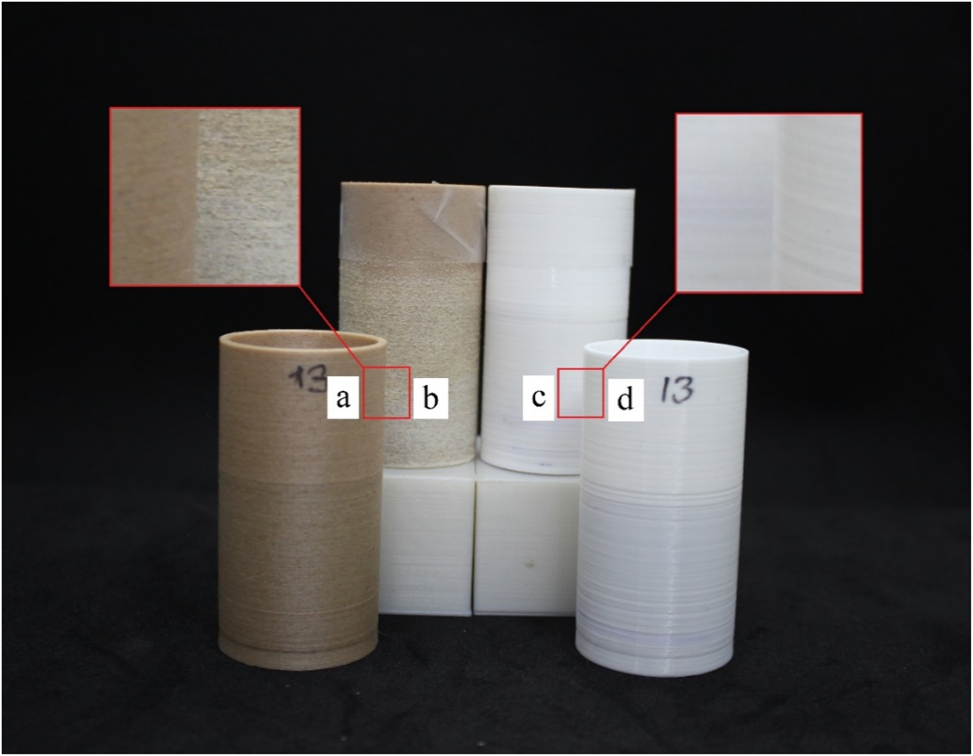
Surface-activated and raw filters; (a) raw PLA/wood composite, (b) surface-activated PLA/wood composite, (c) surface-activated PLA, (d) raw PLA.

**Fig. 5:**
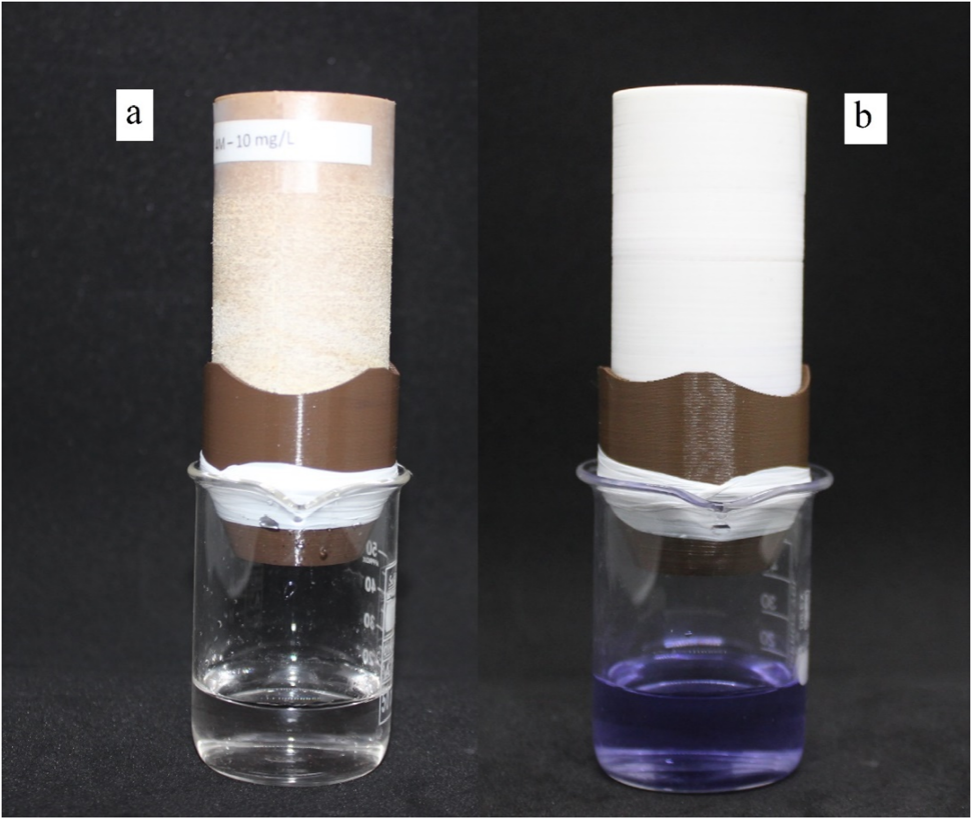
Experimental setup; (a) PLA/wood composite, (b) PLA.

For SEM, XRD, and FTIR tests, 6 × 6 cm plates were produced with PLA and PLA/wood composite filaments using a 3D printer to ensure consistency with the filters. These plates underwent the same KOH activation process as the filters. SEM (Scanning Electron Microscopy) was used to analyze the surface morphology, XRD (X-ray Diffraction) to investigate the crystalline structure, and FTIR (Fourier Transform Infrared Spectroscopy) to identify functional groups on the surface. All thermal treatments were kept below 50 °C to maintain the integrity of the filter material. The total contact time between the dye solution and the filter was controlled to ensure maximum separation. Flow rates were adjusted by modifying the gravity feed setup to maintain consistent exposure times. Preliminary tests indicated that longer contact times improved dye separation efficiency, leading to the standardized contact time used in all experiments. The comparison between the surface-activated and raw filters is shown in Fig. 4, where (a) represents raw PLA/wood composite, (b) represents surface-activated PLA/wood composite, (c) represents surface-activated PLA, and (d) represents raw PLA. The experimental setup for these tests is shown in Fig. 5, where (a) represents the setup with PLA/wood composite, and (b) represents the setup with PLA.

## 3. Results and discussion

**Fig. 6:**
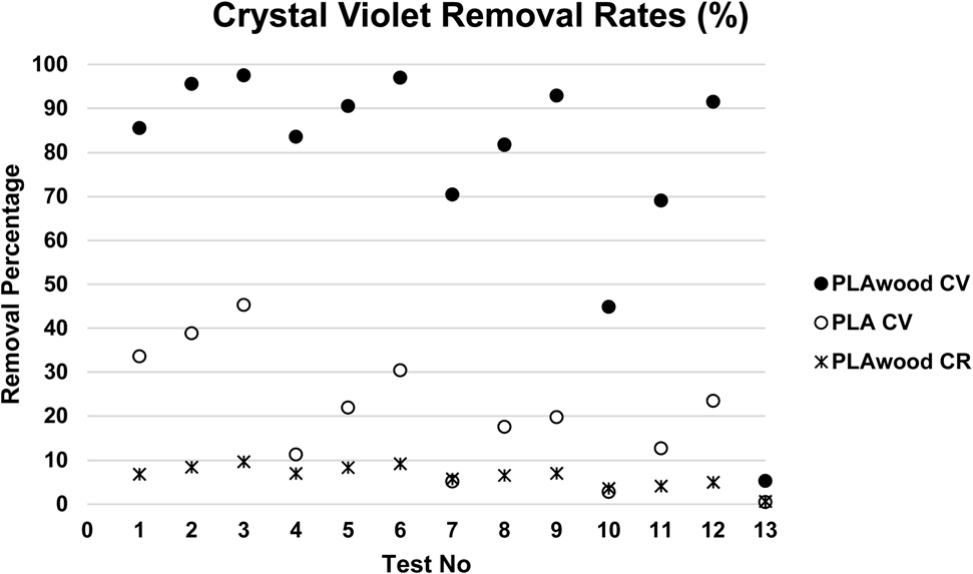
CV and CR removal rates achieved using the designed filter.

The filters designed and produced with 3D printing were tested with solutions of different KOH concentrations (0.1, 0.2, and 0.4 M) and various CV and CR concentrations (10, 25, 100, and 200 mg L^−1^). A 20 mL CV solution was added to the filter housing and allowed to filter by gravity without any additional pressure. The results of the dye removal tests, summarized in Table 2 and Fig. 6.

**Table 2:** Crystal violet and Congo red removal rates (%) based on different test conditions

No.	Inlet color concentration (mg L^−1^)	KOH concentration (M)	Removal crystal violet (PLA, %)	Removal crystal violet (PLA/wood composite, %)	Removal Congo red (PLA/wood composite, %)
1	10	0.1	33.57	85.47	6.81
2	10	0.2	38.82	95.56	8.42
3	10	0.4	45.28	97.5	9.67
4	25	0.1	11.32	83.50	6.99
5	25	0.2	21.89	90.49	8.32
6	25	0.4	30.38	96.94	9.21
7	100	0.1	5.16	70.46	5.74
8	100	0.2	17.49	81.72	6.54
9	100	0.4	19.72	92.85	7.03
10	200	0.1	2.76	44.84	3.56
11	200	0.2	12.71	69.06	4.12
12	200	0.4	23.44	91.45	4.98
13	25	—	0.45	5.28	0.62

The results of control experiments for test 13 show that the PLA/wood composite filter removed approximately 5.28%, while the PLA filter removed only 0.45% of Crystal Violet (CV) from a 25 mg per L CV solution without any processing after 3D printing. The highest filtration rates were achieved with activation using 0.4 M KOH. It was observed that the performance of the filters decreased as the CV concentration increased. The highest removal efficiency was observed in the filtration of a 10 mg per L CV solution using the PLA/wood composite filter activated with 0.4 M KOH, resulting in approximately 97.5% CV removal. For the PLA filter, the highest removal efficiency was 45.28% for the 10 mg per L CV solution with 0.4 M KOH activation.

The removal rates for the PLA filter ranged from 2.76% to 45.28%, while those for the PLA/wood composite filter ranged from 44.84% to 97.5%. These results indicate that filter performance decreased as the CV concentration increased. However, considering that the filter tests were carried out solely under the influence of gravity without any additional pressure, the CV removal rates achieved were notably high.

Electrostatic adhesion has been described in various studies to explain the adhesion of dyes.^40,41^ Interactive forces such as van der Waals, surface tension, and electrostatic forces between micro/nanoparticles and the substrate surface create adhesion.^42^ KOH-based abrasives are preferred due to their lower cost and higher abrasion rate.^43^ It is concluded that KOH activation enhances the filtration capability of the 30% wood additive in PLA/wood composite by modifying the surface properties.

Additionally, 13 of the filters were tested with Congo Red (CR), an anionic dye. Dye removals were measured using the same method applied in the other tests with different KOH and CR concentrations. CR removal efficiencies ranged between 4% and 9%. The comparative color removal percentage graph is shown in Fig. 6.

Wood-filled composite PLA filters can be produced from various types of wood waste, enhancing their environmental sustainability and effectiveness in water filtration. By incorporating a wood additive with high separation properties, PLA/wood composite filters significantly improve the removal efficiency of pollutants from water. Additionally, both the PLA and the wood additive used in these filters are biodegradable, making them environmentally friendly. After the filters have completed their service life, they can be disposed of or repurposed in several environmentally sustainable ways. Composting is one viable option for these biodegradable materials.

### 3.1. SEM images

**Fig. 7:**
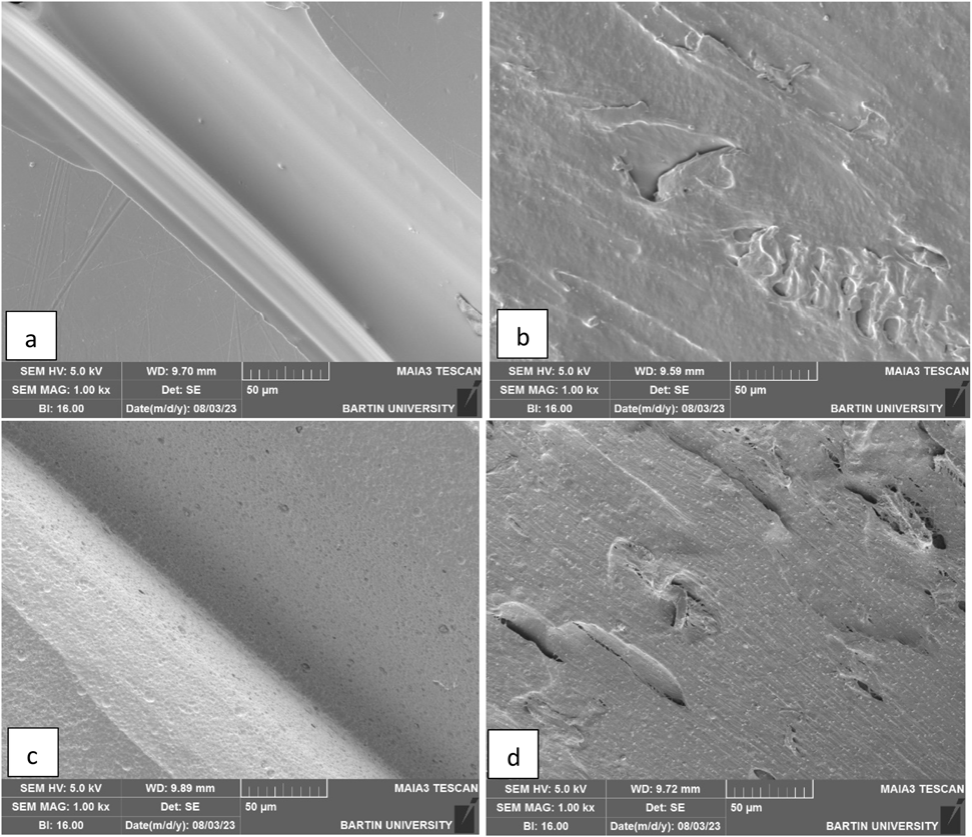
SEM surface images of PLA (a) and PLA/wood composite (b) after printing (×1000), and PLA (c) and PLA/wood composite (d) after four-hour activation with 0.4 M KOH (×1000).

For SEM tests, 6 × 6 cm plates were produced using PLA and PLA/wood composite filaments with a 3D printer, and the same processes were applied to these plates as to the filters. A TESCAN MAIA3 XMU Scanning Electron Microscope was used for SEM imaging. The images of the PLA and PLA/wood composite samples, after 3D printing, 0.4 M KOH activation, and CV removal tests, are shown in Fig. 7.

When examining the surfaces of the PLA and PLA/wood composite samples in Fig. 7, the PLA print appears smooth, while the surface of the PLA/wood composite is rough. During the printing process, the nozzle scrapes the wood additive in its solid form, resulting in a rough surface. This roughness is thought to create more area for the dye to adhere to, providing an advantage for CV removal. Literature studies with composite PLA materials also show rough structures in SEM images after 3D printing.^44,45^

In Fig. 5(c) and (d), cavities and gaps formed on the surfaces of the PLA and PLA/wood composite samples due to 4 hour activation with 0.4 M KOH. The wood composite material in PLA/wood composite resulted in a more abrasive and rough structure during KOH activation. CV filtration was limited in the PLA material due to fewer gaps compared to the PLA/wood composite material. According to the PLA/wood composite images, a much more porous structure was formed after KOH activation. It is concluded that these gaps increase CV removal efficiency.

### 3.2. FTIR analyzes

**Fig. 8:**
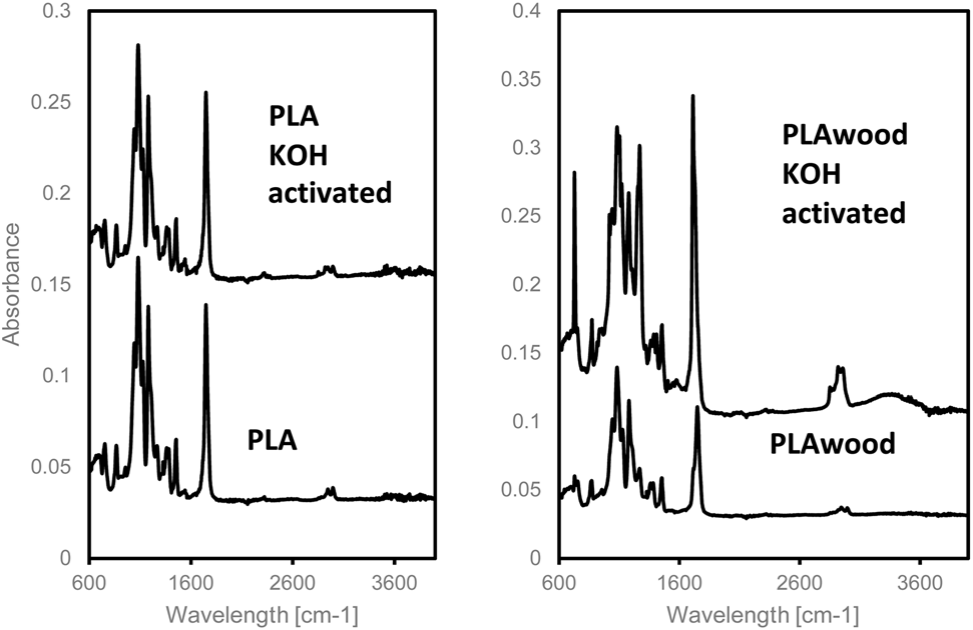
Fourier Transform Infrared (FTIR) spectra of PLA, KOH-activated PLA, PLA/wood composite, and KOH-activated PLA/wood composite.

For Fourier transform infrared (FTIR) spectra tests, 6 × 6 cm plates were produced using PLA and PLA/wood composite filaments with a 3D printer, applying the same processes used for the filters (Fig. 8). The FTIR spectra were obtained using a Shimadzu IRAffinity-1 spectrometer. The FTIR spectra of PLA and PLA/wood composite before and after 0.4 M KOH activation are shown in Fig. 8.

The FTIR spectra of PLA before and after KOH activation show characteristic stretching frequencies for C=O, –CH_3_ asymmetric, –CH_3_ symmetric, and C–O at 1747, 2993, 2945, and 1080 cm^−1^, respectively. Bending frequencies for –CH_3_ asymmetric and –CH_3_ symmetric are identified at 1456 and 1361 cm^−1^, respectively. Activated PLA shows almost the same peaks as raw PLA, suggesting that KOH activation does not alter the structure of the PLA material. Similar results have been reported in the literature. Chieng *et al.* (2014) showed the FTIR spectra of PLA material, with characteristic stretching frequencies for C=O, –CH_3_ asymmetric, –CH_3_ symmetric, and C–O at 1746, 2995, 2946, and 1080 cm^−1^, respectively.^46^ Bending frequencies for –CH_3_ asymmetric and –CH_3_ symmetric were identified at 1452 and 1361 cm^−1^, respectively. The nanocomposite they studied showed the same absorption peak points as pure PLA, indicating no new bond formation or strong chemical interaction in the mixtures and nanocomposites.^46^

When examining the spectra of PLA/wood composite and activated PLA/wood composite, an increase in the peaks of PLA/wood composite after activation was observed. After KOH activation of PLA/wood composite material, the C–H bending peak at 729 cm^−1^ appeared. Additionally, there was a slight increase in C–O stretching bonds at 1083, 1178, and 1271 cm^−1^ peaks. C–H bending is observed at the 1466 cm^−1^ peak.

In studies conducted on composite materials with lignin, it was confirmed that the absorption peaks appearing at 2977 cm^−1^ and 2903 cm^−1^ are the C–H symmetric stretching vibration bands of –NH–CH_2_– and methylene functional groups, respectively.^47,48^ It is thought that the lignin found in the structure of wood waste creates this peak in the PLA/wood composite material.

The FTIR spectrum in the 600–1800 cm^−1^ region shows that KOH activation induces pronounced structural rearrangements in the PLA/wood composite. Most notably, the intensified C=C stretching and aromatic vibration bands centred around ∼1600 cm^−1^ indicate partial cleavage and re-aromatisation of the lignocellulosic matrix, giving rise to a surface that resembles incipient carbonisation. In contrast, the broad absorption that develops in the 2500–3600 cm^−1^ region of the activated sample reflects an enrichment of hydroxyl and/or carboxylate functionalities; KOH promotes ester hydrolysis in the PLA chains and cleaves ether linkages in the lignin–cellulose network, thereby increasing the density of –OH and –COO^−^ groups. The higher surface concentration of these hydrophilic sites facilitates stronger electrostatic attraction toward cationic dyes such as crystal violet, while the partial dissolution of wood components during activation promotes the formation of macro- and mesopores, further enhancing the overall adsorption capacity. Collectively, the FTIR results confirm that KOH treatment not only chemically functionalises the PLA/wood composite surface but also renders it more reactive and hydrophilic, thereby producing a composite that is markedly more effective for cationic-dye removal.

In the absence of thermal treatment, the chemical transformations observed in KOH-treated PLA/wood composite are consistent with the well-documented effects of alkaline activation on both polyesters and lignocellulosic matrices. Hydroxide ions first catalyse the hydrolysis of ester bonds along the PLA backbone, yielding lactate-based oligomers and generating a high surface density of carboxylate (–COO^−^) groups; this alkali-catalysed saponification pathway accords with earlier reports showing that PLA rapidly loses molecular weight in strong alkaline media KOH acts as a strong base in the same medium, cleaving covalent bonds within the cellulose/lignin network, dissolving hemicellulose, and partially depolymerising the aromatic backbone of lignin; FTIR analysis shows a broadening of the –OH band and an intensified aromatic C=C vibration at ∼1590–1600 cm^−1^ after KOH treatment, indicating an increase in –OH/–COO^−^-rich functional domains and partial aromatisation within the lignocellulosic matrix.^49^ Potassium ions, released during the partial dissolution of wood constituents, open macro and mesopores in the cell walls, thereby increasing specific surface area; moreover, K metal/K_2_CO_3_ species embedded in the carbon framework can act as chemical “templates” that promote porosity even in the absence of high-temperature activation.^50,51^ The resulting basic, porous and negatively charged surface strongly adsorbs cationic dyes such as crystal violet *via* electrostatic attraction; the pore network enhances physical retention, while the extended aromatic domains favour potential π–π stacking, collectively boosting overall adsorption capacity.^52,53^ Owing to these multi-modal interactions, KOH-activated PLA/wood composite becomes a hydrophilic, highly functionalised material suitable not only for dye removal but also for ion exchange, catalyst support and energy-storage electrodes. Importantly, no spectral signatures associated with the leuco (reduced) form of crystal violet were observed in either the filtrate or the adsorbed phase, indicating that dye removal was governed by adsorption rather than chemical degradation.

### 3.3. XRD analyzes

**Fig. 9:**
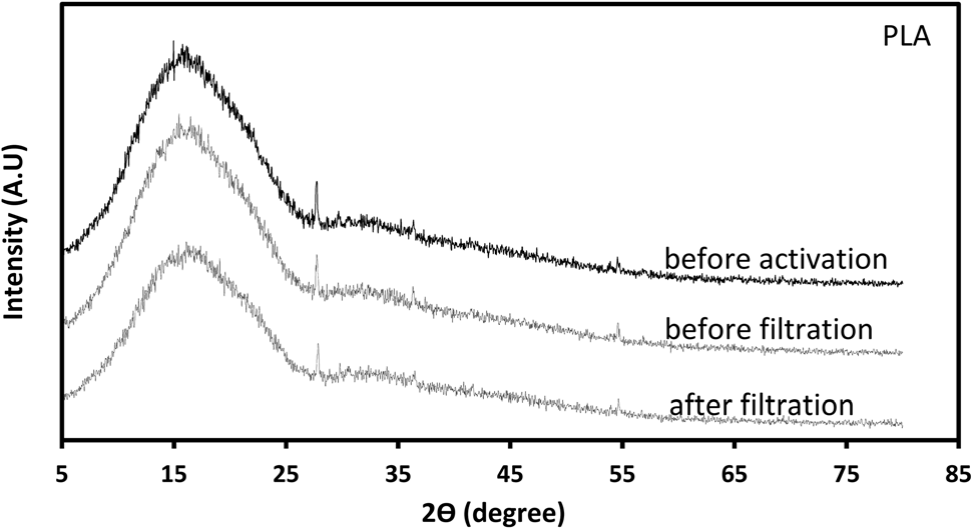
XRD graph of PLA.

**Fig. 10:**
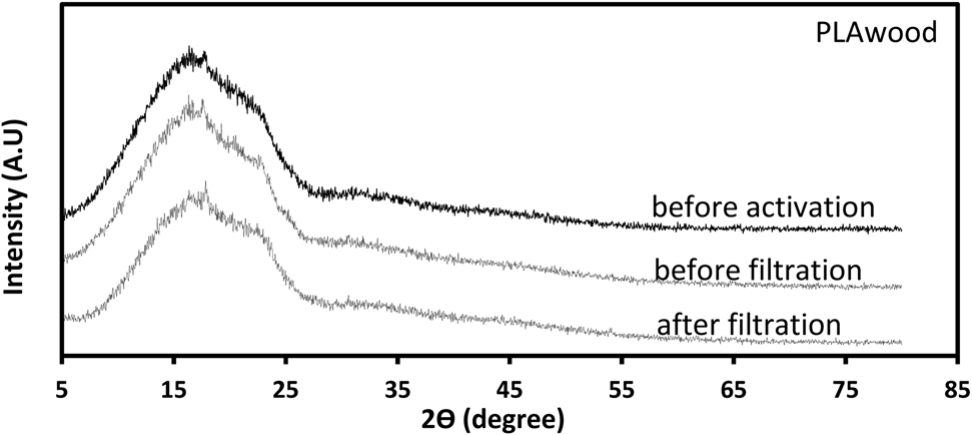
XRD graph of PLA/wood.

XRD measurements were performed using a Rigaku SmartLab diffractometer operating at 40 kV and 30 mA with Cu Kβ radiation. Data were collected in continuous scan mode over a 2*θ* range of 5–80° with a step width of 0.04° and a scan speed of 3° min^−1^. A SC-70 detector, BB incident slit, and 1/2° receiving slit were used. For each sample, 6 × 6 cm 3D-printed plates of PLA and PLA/wood composite were analyzed under the same conditions applied to the filters. Crystallinity was quantified by integrating the crystalline peak area above a linear baseline between 5° and 30° (2*θ*).^54^ The calculated crystallinity indices were 63.17% (PLA/wood composite), 56.74% (PLA), 57.28% and 54.22% for KOH-activated PLA/wood (before/after filtration), and 40.98% and 53.95% for KOH-activated PLA (before/after filtration). These values confirm that both PLA and PLA/wood remain predominantly amorphous,^54^ with only limited crystalline ordering, consistent with the broad diffraction features observed in Fig. 9 and 10.

In the XRD pattern of the neat PLA sample (Fig. 9), two relatively clear diffraction peaks appear at about 2*θ* ≈ 28° and 55°. Based on prior studies,^55–57^ these peaks can be ascribed to the (110)/(203) crystallographic planes of semi-crystalline PLA. In contrast, the PLA/wood composite (Fig. 10) shows significant attenuation of these peaks. This attenuation likely arises from the incorporation of lignocellulosic wood dust, which introduces an amorphous phase and disrupts the regular ordering of PLA chains. Additionally, the KOH activation process may further reduce crystallinity through partial hydrolysis or chain scission. The result is a more amorphous overall structure, consistent with the increased baseline and diminished peak intensities in the composite pattern. For quantitative support, further analysis of full-width at half-maximum (FWHM) and crystallinity index *via* DSC or deconvolution of the XRD peaks is recommended for future studies.

### 3.4. pHpzc analysis

Above the zero charge point (>pHpzc), the negative charge density on the adsorbent surface facilitates the adsorption of basic (cationic) dyes such as methylene blue. Due to the ionic character of the sawdust and the ionic charges on the dyes, the sorption capacity of basic dyes is much higher than that of acid dyes.^8^ The surface of plastics becomes negatively charged when the solution pH > pHpzc.^9^ As the pH of the environment increases, the number of positively charged sites decreases while the number of negatively charged sites increases. Consequently, the surfaces of PLA and PLA/wood composite molecules become more negatively charged as the solution pH increases. These negatively charged areas facilitate the attachment of dye cations due to electrostatic attraction.

In short, negatively charged areas enhance the removal of cationic dyes through electrostatic attraction. This explains the CV removal mechanism in the filter printed with 3D technology and activated with KOH. The practical implications of this mechanism are significant for water treatment applications, where effective dye removal is critical.

**Fig. 11:**
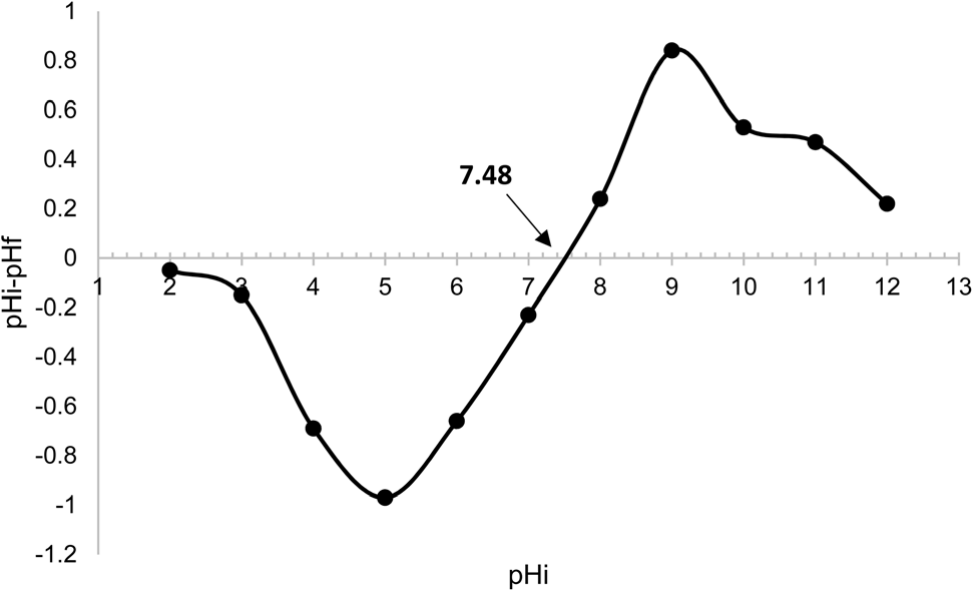
pHpzc graph of PLA/wood composite filter.

The filter material's surface chemistry, neutral charge point, and acid–base neutralization capacities were characterized (Fig. 11). Tests conducted between pH 2 and 12 resulted in a pHpzc value of 7.48 for PLA/wood composite material by interpolation. This value is consistent with findings in the literature; for instance, Lang and Xue (2022) reported a pHpzc value of 7.3 for PLA material.^58^ Shukla *et al.* (2002) found similar pHpzc values for various sawdust materials: polyacrylamide-grafted sawdust at 5.9, sawdust treated with dye at 5.9, sawdust activated carbon at 6.4, sawdust treated with polysulfide at 4.3, and rubber wood sawdust carbon at 5.8.^59^ Batzias and Sidiras (2007) measured the pHpzc value of beech sawdust to be 5.2 ± 0.2.^8^ Taken together, the SEM, FTIR, and pHpzc results confirm that KOH activation produces a negatively charged, hydroxyl- and carboxyl-rich surface that binds cationic dyes electrostatically. The observed dye removal thus originates from physical adsorption and electrostatic attraction, not from any chemical transformation of the dye molecules.

### 3.5. Regeneration tests

**Fig. 12:**
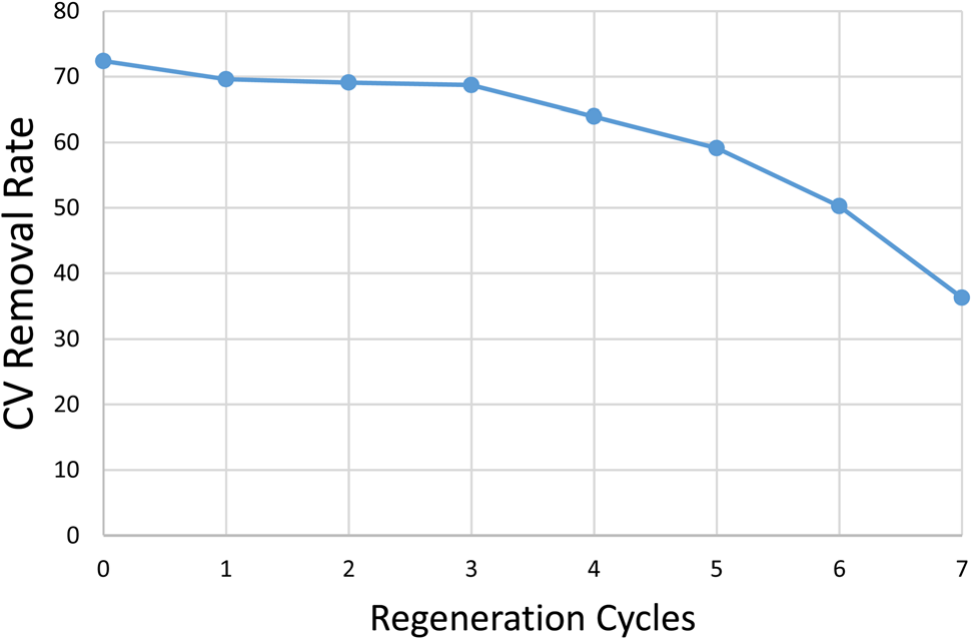
CV removal *versus* regeneration cycles.

Although regeneration was not the main focus of this study, a preliminary test was conducted to assess the feasibility of filter reuse under simple acid-washing conditions. Further optimization of regeneration chemistry was beyond the present scope; the test was included only to evaluate the material's basic reusability. The study aims to produce cost-effective water treatment filters for dye removal that can be used in regions with limited resources. Therefore, the economic aspect of such filters is essential. In this context, the potential for reuse of this filter has also been tested. The economic viability of the KOH-activated PLA/wood composite filter was assessed through sequential adsorption–desorption cycles using 0.5 M HCl as regenerant. After each run, the filter was rinsed three times with de-ionised water and dried at 45 °C for 72 h. Crystal violet (25 mg L^−1^) removal remained essentially unchanged during the first three cycles, falling only from 72% in the pristine material to 69% after the third regeneration. Beyond this point, performance declined steadily—64% (cycle 4), 59% (cycle 5), 50% (cycle 6) and 37% after the seventh cycle (Fig. 12)—equivalent to ≈18% cumulative loss after five full cycles and ≈35% over the entire seven-cycle series. Although these values still compare favourably with many low cost sorbents reported in the literature, they indicate that the present regeneration protocol is sustainable for roughly three reuse cycles before significant efficiency loss sets in.

The deteriorating trend is attributed to two coupled phenomena. First, HCl neutralises residual KOH (KOH + HCl → KCl + H_2_O) and protonates the alkali-generated –COO^−^/–OH sites, converting them into less reactive –COOH groups and lowering the surface's negative charge; the accompanying pH drop also accelerates acid-catalysed hydrolysis of PLA chains and partial depolymerisation of hemicellulose, subtly altering pore morphology. Second, incomplete dye removal together with precipitated KCl progressively blocks micro- and mesopores, restricting diffusive access to active regions. These effects jointly diminish both the number and accessibility of adsorption sites, explaining the marked efficiency loss beyond cycle 4. To prolong service life in resource-constrained settings, milder acidic eluents, periodic re-alkalinisation, or alternative regeneration chemistries that restore –COO^−^/–OH density without degrading the matrix are recommended.

### 3.6. Performance comparison of different materials

Filters of identical design were fabricated under identical conditions using commercially available PLA, PLA–GF containing 30% graphite (Filameon®), and PLA/CF containing 15% carbon fiber (Filameon®) commercial filaments, as well as a PLA/wood composite material containing 30% wood powder (Porima PLA®). Graphite and carbon fiber are known for their high surface area and adsorption capabilities, while wood powder introduces natural porosity and potentially beneficial chemical interactions.

**Fig. 13:**
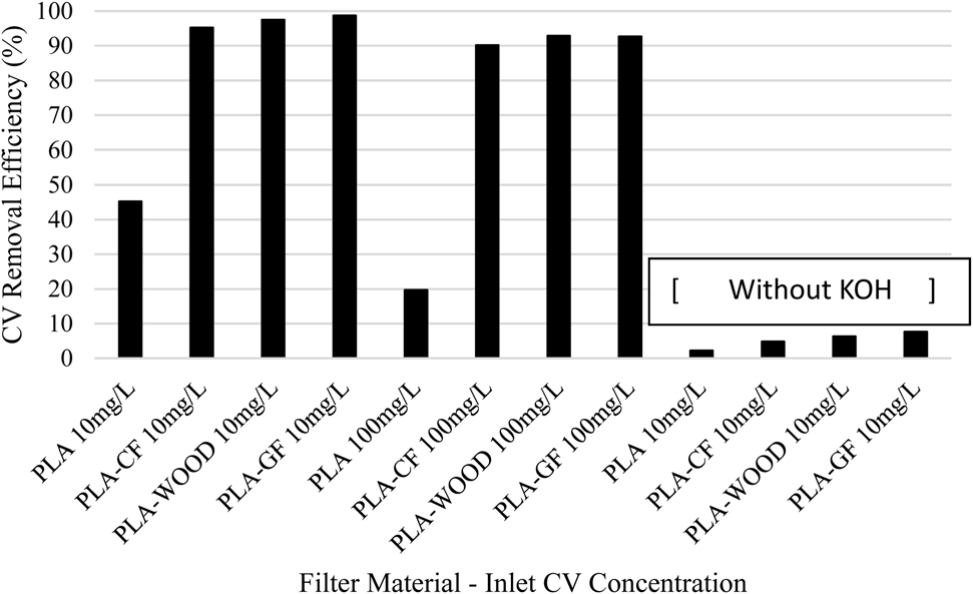
CV removal rates based on different test materials.

The removal performance of the cationic dye crystal violet was investigated using these PLA-based biodegradable materials (PLA, PLA/CF, PLA/wood composite, and PLA/GF), both in their raw form and after activation with 0.4 M KOH (as described in Section 2.1). The CV removal rates under various test conditions are summarized in Table 3, presenting the removal efficiencies (%) for different materials and scenarios. As shown in Fig. 13, the CV removal efficiency varies significantly depending on the type of material used in the filter.

**Table 3:** CV removal rates (%) based on different test materials

Filter material	Dye concentration (mg L^−1^)	Removal efficiency (%)
PLA–KOH	10	45.28
PLA/CF–KOH	10	95.24
PLA/wood–KOH	10	97.5
PLA/GF–KOH	10	98.72
PLA–KOH	100	19.72
PLA/CF–KOH	100	90.19
PLA/wood–KOH	100	92.85
PLA/GF–KOH	100	92.68
PLA	10	2.25
PLA/CF	10	4.85
PLA/wood	10	6.35
PLA/GF	10	7.65

Among the various PLA-based materials, PLA/wood composite and PLA/GF demonstrated the highest removal performance, achieving 97.5% and 98.72% removal efficiency, respectively. The superior performance of PLA/GF can be attributed to the high surface area and adsorption capacity of graphite. Similarly, the wood powder in PLA/wood composite likely contributed to its high performance by increasing the surface area and potentially introducing favorable electrostatic interactions with the dye. The carbon fiber-reinforced PLA/CF material also performed well with a 95.24% removal efficiency, but it lagged slightly behind the wood- and graphite-reinforced materials.

This study demonstrates the potential of 3D printing technology for fabricating high performance filters using various PLA-based materials. The incorporation of additives like graphite and wood powder significantly enhances the dye removal efficiency. Notably, the PLA/wood composite material offers a promising sustainable alternative due to its use of natural additives and inherent biodegradability. Further research exploring alternative designs, material compositions, and performance evaluations in this field is crucial to fully realize the potential of 3D printing for water purification.

### 3.7. Limitations and future outlook

While the present work demonstrates the strong potential of KOH-activated PLA/wood filters for cationic-dye removal, several aspects remain to be explored. The study is limited by gravity-driven operation, testing only three KOH concentrations and two dyes, and using a single filter geometry. Regeneration was assessed with one acid protocol and only seven cycles. Mechanical durability, long-term use, and broader pollutant applicability were not evaluated.

## 4. Conclusions

This study demonstrated that KOH activation substantially enhances the performance of 3D-printed PLA/wood composite filters for cationic dye removal. The PLA/wood material activated with 0.4 M KOH achieved the highest crystal violet removal, reaching 97.5% at 10 mg L^−1^ under gravity-driven conditions. Across all concentrations, PLA/wood composites outperformed neat PLA, consistent with SEM observations showing increased surface roughness and FTIR results indicating a higher density of –COO^−^ and –OH groups after activation. XRD analyses confirmed that both materials remained predominantly amorphous before and after treatment. Congo red removal efficiencies were low, highlighting the selectivity of the activated composite toward cationic dyes. Overall, the findings confirm that material composition and alkaline surface modification are key determinants of dye removal efficiency in 3D-printed PLA-based filters.

## Author contributions

The author was solely responsible for the following aspects of the research: conceptualization, methodology, investigation, data curation, formal analysis, writing – original draft, and writing – review & editing.

## Conflicts of interest

There are no conflicts to declare.

## Supplementary Material

RA-015-D5RA08506C-s001

## Data Availability

All data necessary to evaluate the conclusions of this study are contained within the article and its supplementary information (SI). No additional data are available. Supplementary information is available. See DOI: https://doi.org/10.1039/d5ra08506c.
